# Institutional tensions, corporate social responsibility and district-level governance of tobacco industry interference: analysing challenges in local implementation of Article 5.3 measures in Karnataka, India

**DOI:** 10.1136/tobaccocontrol-2021-057113

**Published:** 2022-01-25

**Authors:** Praveen Kumar, Rachel Ann Barry, Muralidhar M Kulkarni, Veena Ganesh Kamath, Rob Ralston, Jeff Collin

**Affiliations:** 1 Department of Commerce, Manipal Academy of Higher Education, Manipal, Karnataka, India; 2 Global Health Policy Unit, University of Edinburgh School of Social and Political Science, Edinburgh, UK; 3 Department of Community Medicine, Kasturba Medical College, Manipal Academy of Higher Education, Manipal, Karnataka, India; 4 Spectrum Research Consortium (Shaping Public Health Policies to Reduce Inequalities and Harm), University of Edinburgh, Edinburgh, UK

**Keywords:** tobacco industry, low/middle-income country, public policy

## Abstract

**Introduction:**

Accelerating progress on tobacco control will require Article 5.3 of the WHO Framework Convention on Tobacco Control to be systematically integrated into policies and practices of sectors beyond health at diverse government levels. However, no study has explored implementation challenges of Article 5.3 within multilevel systems such as India, where political decisions on tobacco control occur at diverse government levels, which may constrain action at local level.

**Methods:**

Based on 33 semi-structured interviews with diverse government and civil society stakeholders across four districts in Karnataka, India (Mysore, Mangalore, Bengaluru (rural) and Udupi), this study examines challenges to implement Article 5.3 arising from competing agendas and policies of different actors at multiple levels.

**Results:**

Our analysis reveals generally low levels of awareness of Article 5.3 and its guideline recommendations, even among those directly involved in tobacco control at district level. Efforts to implement Article 5.3 were also challenged by competing views on the appropriate terms of engagement with industry actors. Scope to reconcile tensions across competing health, agriculture and commercial agendas was further constrained by the policies and practices of the national Tobacco Board, thereby undermining local implementation of Article 5.3. The most challenging aspect of Article 5.3 implementation was the difficulties in restricting engagement by government officials and departments with tobacco industry corporate social responsibility initiatives given national requirements for such activities among major corporations.

**Conclusions:**

Promoting effective implementation of Article 5.3 in Karnataka will require policymakers to work across policy silos and reconcile tensions across India’s national health and economic priorities.

## Introduction

Accelerating progress on tobacco control requires Article 5.3 of the WHO Framework Convention on Tobacco Control (FCTC) to be systematically integrated into the policies and practices of sectors beyond health within and across government levels.^1 2^ As a general obligation, Article 5.3 of the WHO FCTC requires that Parties act to protect public health policies from the commercial and other vested interests of the tobacco industry in accordance with national law.^3^ Despite widespread recognition of its foundational status for the FCTC^4^ and as the key catalyst for further progress in international tobacco control,^5^ country-level implementation of Article 5.3 remains poor, including in many low/middle-income countries.^2 6^

India represents an important context in which to examine the challenges of Article 5.3 implementation, particularly given the significance of subnational actions. In 2020, India’s Ministry of Health and Family Welfare adopted a code of conduct to protect tobacco control policies and programmes from industry interference, the scope of which is limited to ministry officials.^7^ This followed introduction of measures to implement Article 5.3 by 13 states and union territories from Punjab in 2015 to Karnataka, Kerala, Uttar Pradesh and Meghalaya in 2019.^8^ Within these diverse initiatives, Karnataka’s experience is distinctive in that the adoption of a state-level notification followed a series of initiatives taken by eight districts (while 14 districts in West Bengal have issued guidelines, these have not led to state-level action).^9^ Karnataka therefore offers a timely case study for examining challenges and opportunities of implementing Article 5.3 at local level, addressing the dearth of studies of subnational FCTC implementation. This is a potentially significant gap given that actions across diverse local venues have afforded important opportunities to circumvent industry opposition in advancing tobacco control.^10–13^

Karnataka is located in India’s southwest and, with a population of around 61 million, is its ninth largest state.^14^ The rationale for Karnataka as a case study for Article 5.3 implementation also encompasses the state’s significance to both tobacco control activism and to tobacco production in India. The genesis of India’s code of conduct for public officials to prevent industry interference can be traced back to a 2010 decision of the High Court of Karnataka requiring its development, following litigation brought by public health activists to challenge governmental participation in an industry event.^15 16^ Alongside this, Karnataka occupies an important position within India’s tobacco industry; India ranks third in global production and fourth in exports^17 18^ while Karnataka is its fourth largest tobacco-producing state.^19^

States and districts have the opportunity to play significant roles in tobacco control given divided responsibilities across the multiple levels of India’s federalised government system. While national tobacco control is centred on the 2003 Cigarettes and Other Tobacco Products Act (COTPA), from which the absence of measures to address Article 5.3 is a key omission,^20^ state legislatures have authority to legislate on public health issues, with restrictions on tobacco sales generally being governed by state laws. In Karnataka, the State Tobacco Control Cell (STCC), developed in 2004 under the Department of Health and Family Welfare, is responsible for implementation and monitoring of state tobacco control activities. Key functions around implementation and enforcement of tobacco control policies and regulations have been further devolved to 30 (totalling 31 as of 2021^21^) newly created District Level Coordinating Committees (DLCCs).^22^ These local multisectoral platforms are designed to coordinate action across multiple departments, including health, agriculture, revenue, law enforcement, administration and education. In this institutional context, between 2017 and 2018, 8 of Karnataka’s 30 district administrations issued notifications to advance implementation of Article 5.3 measures^23 24^; these actions were followed, and superseded by, the Karnataka-wide notification issued by the STCC in 2019.^25^

Both state and district notifications address key elements of WHO Article 5.3 implementation guidelines^26^ focused on limiting government-industry interactions and promoting transparency in those that occur.^23–25^ While state policy expands on district-level action in specifying a code of conduct for all public officials, it omits reference to tobacco industry corporate social responsibility (CSR) initiatives. Udupi and Bengaluru (rural), by contrast, prohibit district government officials and employees from accepting ‘any direct/indirect/in-kind sponsorship/donation/funding in [the] form of CSR support’ from tobacco industry actors. This is an important point of distinction given the ongoing significance of tobacco industry CSR in Karnataka and across country,^27 28^ where policy tensions are shaped by India’s Companies Act requiring large businesses (including tobacco companies) to allocate 2% of net profits to CSR actions.^29^

Based on semi-structured interviews with key informants, this paper explores district-level challenges involved in seeking to implement Article 5.3 across four districts in Karnataka. It first examines varying levels of awareness of and engagement with Article 5.3 measures across officials in health and other departments, and highlights contrasting perspectives on the appropriateness of government interactions with the tobacco industry. Contestation in managing industry interference is then explored with reference to institutional conflicts and competing mandates across government actors, centred on the role of the national Tobacco Board, and to policy tensions surrounding efforts to limit government engagement in tobacco industry CSR.

## Methods

We conducted a qualitative analysis of implementation challenges across four districts in Karnataka: Bengaluru (rural), Udupi, Mysore and Mangalore. These were selected to cover variation in policy routes to managing industry interference and in the local economic significance of tobacco. Bengaluru (rural) and Udupi both issued district-level notifications in 2017, while Mysore and Mangalore only became subject to Article 5.3 measures via state-level action in 2019. The tobacco industry does not have a notable presence in the two chosen districts that issued notifications, while Mysore is Karnataka’s largest tobacco-growing district^30^ and the beedi industry has a long established presence in Mangalore.^31^

Tobacco control in Karnataka is comprised of three different levels: state, district and taluk (an administrative subdivision of district governments).^20^ DLCCs, situated under the Deputy Commissioner, have responsibility over implementation of tobacco control policies and programmes.^22^ While acknowledging the existence of lower levels of government in Karnataka, extending down to village level, we are focusing on district-level efforts, as the DLCC plays a key role in advancing Article 5.3 implementation.

Reflecting this, we explored challenges in implementing measures to manage industry interference via semi-structured interviews (n=33) with members of DLCCs (n=24) and state-level officials (n=7) from diverse government departments, and with tobacco control researchers in India. We approached 39 potential interviewees, of which 33 agreed to be interviewed for this project. The relatively high acceptance rate (33 of 39=85%) suggests that those engaged in tobacco control at district and state level in Karnataka were generally interested in speaking about their experiences and perceptions.

Interviewees were purposively selected based on their involvement in or knowledge of tobacco control issues in the four districts, with particular emphasis on recruiting participants in DLCCs and at state level. Our sample includes more participants from health departments than other government sectors, reflecting varying levels of engagement with tobacco control and heightened challenges of recruiting participants during the COVID-19 pandemic. Table 1 summarises the distribution of interviewees by location and by role, with these separated to maintain anonymity.

**Table 1:** Interviewees by location and role

Location	Number
Mysore	8
Mangalore	7
Bengaluru (rural)	5
Udupi	6
State level	7
**Total**	**33**
**Role**	**Number**
Health	14
Public administration	2
Agriculture	3
Education	5
Police	3
Revenue	3
Researchers	3
**Total**	**33**

Interviews were conducted between July 2020 and April 2021 in person (n=19) or via telephone (n=14) by PK, either in English (n=22) or Kannada (a regional language in Karnataka; n=11) according to interviewee preference. Interviews were semi-structured, employing an interview schedule topic guide organised around four key themes: awareness of FCTC Article 5.3 and its guideline recommendations; approaches to interaction or collaboration between government and the tobacco industry; perceptions of barriers and facilitators for coordinated implementation across government departments and levels; perceptions of tobacco industry interference and CSR. Interviews ranged from 15 to 90 min, with an average duration of approximately 25 min. All interviewees provided consent to participate in the study, while seven preferred not to be audio recorded; in those cases, extensive notes were taken during the interview and used as background information but are not directly quoted. Interviews were transcribed and translated verbatim into English by PK and a research assistant, and coding was undertaken using NVivo V.12 software by PK and RAB. This involved thematic analysis using an iterative process, in which codes and subcodes were developed inductively through repeated readings of the transcripts. Coded data were then used to develop a narrative analysis, comparing facilitators and barriers to implement Article 5.3 within the selected districts of Karnataka.

## Results

### Awareness of Article 5.3

Our results are organised around four key themes that emerged from the data analysis. These include: (1) different levels of awareness of Article 5.3; (2) contrasting understandings of government-industry interactions; (3) institutional tensions across health and agriculture; and (4) the challenges to regulate CSR amid policy tensions.

The interview data indicate broad familiarity with the FCTC and with Article 5.3 across health officials engaged in tobacco issues at state and district levels. Industry interference issues were a prominent focus for district committees, with health officials reporting that ‘Article 5.3 is one of the common agendas in all DLCC meetings’ and that ‘whoever attended those meetings are aware of it’. This prominence was reflected in awareness-raising activities for government officials, such as workshops conducted by health officials in Bengaluru with ‘all district and taluk (subdistrict) officers’ and in Udupi as part of having ‘continuously trained all government officials and strongly emphasised the concept of the FCTC’. Experience gained with district-level approaches was also used to build awareness among state officials, facilitating Karnataka’s 2019 notification. As one health official noted:

As a divisional coordinator, I conducted many meetings, one-to-one meetings to create awareness, sensitise at district-level. When it comes to the state-level, I shared my experience handling the issues at district-level. We informed all other officers what the necessity is of implementing this in all districts. And when the discussion came in [the] High Power Committee, it was possible to bring the state protocol to state-level.

Yet, despite such activities, awareness of commitments under state and district notifications across non-health departments seemingly remained low, with interviewees from education and administration sectors reporting neither knowledge of the FCTC nor having heard of Article 5.3. One health official experienced in such dissemination activities reported how colleagues in other state departments ‘were very surprised to know that something like this exists. They are also unaware of the notification’. Another tobacco control researcher, however, expressed scepticism about claimed ignorance, which could be convenient in avoiding new obligations. This interviewee suggested that some colleagues in other departments:

…are aware but acting like they are unaware. They are clever people. What you call ‘Bend it like Beckham’; I use that phrase with these government officials. To bend it like Beckham is to accommodate the Tobacco Board and the politicians.

Yet even among some health officials working in DLCCs, there was uncertainty and ambiguity about specific provisions for limiting engagement with the tobacco industry. Two officials working in different districts asserted that ‘there is no such restriction’ and that ‘[n]o one has instructed us not to interact with tobacco company people’.

### Contrasting understandings of government interactions with the tobacco industry

Interview data indicate that health officials engaged in tobacco control across state and district levels broadly endorsed Article 5.3 restrictions on engagement with the tobacco industry. In Mysore, for example, health officials identified such government-industry interactions as necessarily entailing a ‘conflict of interest’ for policymakers, reflected in preferential treatment for the tobacco industry (such as price and market supports and in the operation of the Tobacco Board’s compulsory auction system). In this view, such practices were inconsistent with the FCTC ‘prohibit[ing] interactions between the tobacco industry and government officials at all levels’.

This endorsement of principles underpinning Article 5.3 was reflected in health officials displaying awareness of (and active engagement with improving) practices to manage industry interference and to promote transparency. A decision-maker involved in implementing tobacco control measures at district level noted that if government officials ‘have to meet any industry person, they should get approval from the health commissioner’, while one district-level consultant claimed that ‘except the health commissioner, no one should interact with tobacco industry people’. Members of the STCC similarly noted requirements that any meetings with industry representatives should be conducted ‘in a public forum at a government office with minutes documented on what they [tobacco companies] want to know’ and that there should be ‘a clear agenda’ for appointments with company representatives. One tobacco control researcher highlighted opportunities to more effectively restrict interactions through minor additions to administrative practices:

Whenever officials receive emails or phone requests for a TI meeting, keep a small checklist verifying from which company or industry they are from and what they want to talk about. The small measures can help avoid those interactions altogether; rather than confirming an appointment, the official realises that this person is from the industry.

There was a marked contrast in perspectives on such interactions between those working in health and other sectors. All district-level representatives from tax, administration and agriculture sectors, and some from police departments, were sceptical about the appropriateness or necessity of limiting their interactions with the tobacco industry. These officials contended that such interactions were entirely normal and that tobacco companies should be treated ‘equally’ as any other business or stakeholder. One district-level tax official characterised their departmental practices as open to:

Any stakeholder for that matter, it may be a taxpayer, a consultant, or through trade bodies like the Chamber of Commerce. Accountants. Everybody has the liberty to interact with us, take our suggestion with respect to the law and its implications. Everybody has ample opportunity to approach our department at any time.

One tax official did partially acknowledge that distinctive implications arose from tobacco industry interactions with government. By contrast with the whole-of-government scope of Article 5.3 and state-level and district-level notifications, however, these interactions were identified as restricted to particularly ‘sensitive’ sectors and as irrelevant to interactions with tax officers:

As far as our department is concerned, we don’t deal with tobacco products in any different manner. Maybe other departments like health and revenue, considering the public health and overall nature of the product, they might be having some sensitivity towards it, but we deal with [*all*] commodities in similar manner.

### Institutional tensions across health and agriculture: the Tobacco Board

The significance of such varying perspectives and of contrasting institutional interests and mandates is evident in how tensions between health and agriculture priorities play out in Karnataka. From the perspective of district officials, these centred on the structure and role of the national Tobacco Board in promoting and protecting tobacco producers’ interests. When asked about priorities for strengthened implementation of Article 5.3 and tobacco control, one district health official suggested:

They must abolish the Tobacco Board, which encourages farmers to grow more. Every year, a policy decision must be made to reduce crop size by 10%. Similarly reduce acreage by 20% and do not give subsidies to [tobacco] farmers.

Confusion across policy objectives was also acknowledged within agricultural policy, with one district officer in that department noting that the ‘government is supporting tobacco through the Tobacco Board. The same government is approaching us to promote alternative crops’.

Alongside horizontal differences across policy sectors, these tensions also reflected a vertical dimension across levels of government. India’s constitution designates tobacco growing functions—particularly implementation of tobacco licensing and auctioning policies and regulations—to the Tobacco Board, affording it national responsibilities that can appear incompatible with district-level and state-level policies. According to one state agriculture official:

Tobacco is grown under the control of the Tobacco Board. They issue licences for these barrens…The Tobacco Board purely does that. There is no role of the agriculture department in it. It is not under our purview at all. It’s purely under central government and the Tobacco Board takes care of it.

The Tobacco Board’s provision of subsidies and other forms of financial support for tobacco agriculture was seen as a significant barrier to advancing tobacco control within Karnataka. One state health official noted the Tobacco Board’s role in setting targets for tobacco crops, and described the government’s role in marketing and auctions as ‘one kind of violation of Article 5.3’ requiring multisectoral action. In a similar vein, another state tobacco control official noted the need for improved coordination across health and agriculture officials and between state and national levels:

Tobacco Board is giving subsidy. We are working to reduce that subsidy. They are reducing the tobacco cropping target every year. But it is not happening as such due to tobacco industry interference. So, I think we have to deal with that particular aspect in collaboration with the Department of Agriculture and Horticulture and the central government.

### Regulating CSR: contestation and uncertainty amid policy tensions

Interview data indicate that difficulties in restricting engagement by government officials and departments with tobacco industry CSR initiatives were experienced as the most challenging aspect of Article 5.3 implementation in Karnataka. One state health official described securing agreement to remove one tobacco company’s logo from a local college sponsored by the tobacco conglomerate as having been ‘really a herculean task’ amid reluctance to view such support as industry interference or as infringing FCTC commitments.

The data do not indicate a clear divide between health and other officials in perceptions of tobacco industry CSR. There was considerable support for policies of non-engagement across diverse government agencies. One district official in administration reported that interactions were discouraged within a department in which ‘we are careful not to use such funds for government purposes’. In one district, an education official described such industry initiatives as a form of ‘indirect promotion[al]’ activity. One counterpart in another district highlighted conflicts of interest and potential to undermine health promotion among students given that ‘the money they sponsor is the money they earned from selling cigarettes’. A police official posed this more starkly: ‘How can we accept such money? They are killing people.’ Conversely, a minority of health officers regarded engagement with tobacco industry CSR as unproblematic, as in this district official advocating that district governments should accept such funding: ‘[These companies] pay crores of rupees as tax to government. [Tobacco company] itself pays in crores every day. If they sponsor [us] through CSR, let’s take it. What’s wrong with that?’

This willingness to countenance such funding illustrates what one state health official refers to as a ‘lacuna’ in adherence to Article 5.3 in which ‘when any activity comes under CSR, we don’t see it as a FCTC violation.’ In part, this reluctance to preclude engagement with CSR initiatives appears driven by resource constraints. One state education official presented limited funding for school infrastructure as requiring local government to accept tobacco industry support:

The government is not stopping the revenue that they receive from such products because it is unhealthy—same way for the betterment of the kids. There is nothing wrong with accepting such sponsorship.

More broadly, however, interview data highlighted tensions between the objectives of Article 5.3 to regulate voluntary initiatives by the tobacco industry and a national policy context promoting CSR by large corporations. The obligation under India’s Companies Act 2013^29^ that such businesses allocate 2% of average net profits to CSR activities was described by one interviewee as negating any sense of conflict of interest across government departments: ‘they feel it is ok as any other industry and CSR is legitimate per Indian legislation’.

The uncertain engagement with Article 5.3 requirements is illustrated by the ambiguous and uneven response to one tobacco company’s ‘Wealth out of Waste’ initiative, focused on joint environmental initiatives the company signed with several city councils in Karnataka including Mysore.^32–34^ Despite considerable opposition from the STCC, including citing incompatibility with COTPA, officials in the administration department were generally seen as supporting such engagement with the industry. This was explained by one tobacco control consultant as resources being ‘given under CSR activity and nothing is wrong with giving them [the tobacco industry] responsibility over solid waste management’. While the agreement between Mysore and the tobacco company was technically cancelled in 2019, it was apparently revived after it had switched to delivering the programme via a local non-governmental organisation (NGO). One STCC representative described having ‘found out that they are doing waste disposal work through an NGO indirectly with the government’. Yet some tobacco control officials were willing to support industry CSR initiatives following such changes in format.^34^ One state health official claimed that the revised waste initiatives were compatible with Article 5.3 obligations once industry imagery had been removed:

An agreement happened. It happened through some other NGO. And it was instructed not to use tobacco company logos or names anywhere. They agreed to this. Our intention was to stop the direct promotion of tobacco companies to the public. According to Article 5.3 of the FCTC, this type of agreement should not be there with a tobacco company. We feel that intention was fulfilled.

## Discussion

In outlining barriers encountered in district-level approaches to managing tobacco industry interference, the interview data presented above vividly illustrate how challenges of FCTC implementation in general and Article 5.3 in particular are defined by routine, local experiences of everyday work to advance tobacco control. Rather than being confined to the remote sphere of international relations and national-level obligations incurred via treaties,^35–37^ disputes and controversies presented here centre on issues of funding for local schools or municipal waste management contracts.

The data suggest that district-level and state-level notifications have already achieved moderate success in reshaping government-industry interactions. Consistent with previous analyses of Article 5.3 implementation in the European Union,^38^ the process of adopting such policies appears to have had an agenda setting function, with issues of industry interference regularly addressed by DLCCs and the STCC. Importantly, this engagement extends to Mysore and Mangalore, notwithstanding significant local interests in tobacco production. There are also some indications of policy success, although hard won, with the removal of a tobacco company’s logo from a local school indicating that notification has buttressed efforts by health officials and advocates to address government-industry engagement. The interview data also suggest that district-level experiences informed the subsequent development of Karnataka’s state-level notification, though further research is required to explore in more detail the interplay between local and national dynamics^8^ and variations between the content of the state’s policy and those adopted in Bengaluru (rural) and Udupi.

The evidence of broad engagement and support for Article 5.3 among health officials significantly exceeding those among other departments largely replicates at local level a familiar pattern from international studies.^2 38 39^ Interesting variations on it are evident, however, with respect to engagement with tobacco industry CSR. On the one hand, there is significant acknowledgement of associated conflicts of interest among officials in diverse government departments, including administration, education and police. Conversely, however, health officials were not unanimous in their opposition to government departments collaborating with industry CSR programmes, amid reticence about seeing its regulation as part of a wider agenda of minimising tobacco industry interference.

Alongside justification based on the need to access an available source of funding in resource-constrained contexts, the data highlight the importance of policy tensions between local efforts to counter tobacco industry CSR and the wider legitimation provided by India’s Companies Act. Within Karnataka, their ongoing salience is illustrated by the state’s Revenue Department accepting a donation from one tobacco company’s education trust for COVID-19 relief efforts, which received public appreciation from the Chief Minister’s office ‘for their generous donation’.^28 40^ This is consistent with reports on the ongoing significance of CSR as a mechanism of tobacco industry interference in India, heightened in the context of COVID-19.^15 28^ Viewed at a national level, this indicates a need for the process of amending COTPA to tackle tensions with the Companies Act and to promote effective Article 5.3 implementation.^41^

Internationally, these context-specific policy tensions have broader relevance, given the lack of coherence between Article 5.3 measures and international commitments to CSR, collaboration and voluntarism in global health and sustainable development.^1 42^

Alongside tensions arising from specific policies, the interview data clearly indicate the extent to which efforts to manage industry interference are circumscribed by institutional conflicts of interest.^43^ Centred in particular on the structure of the Tobacco Board and its role in protecting and promoting producer interests, accounts from multiple officials highlight how conflicting mandates recognised at national level^15 27^ impact on practices at district and state levels. The absence of any measures to address Article 5.3 implementation guidelines around avoiding preferential treatment of tobacco industry interests constitutes notable omissions across district and state notifications under examination. Such institutional tensions have important territorial components, given the Tobacco Board’s national mandate and local remit of the district committees and the STCC. Future research could usefully explore strategies for mitigating and managing such conflicts^44^ and improving communication on implementation across national and subnational levels of government as well as across policy spheres.

## Conclusion

This study demonstrates the significance of district-level innovation in the adoption and spread of measures to implement Article 5.3, alongside highlighting significant barriers to be addressed. It illustrates the importance of moving beyond the national level in exploring opportunities to advance FCTC implementation in both research and policy contexts, emphasising the potentially strategic advantages of local initiatives in seeking to minimise tobacco industry interference.

**What this paper adds**

**What is already known on this subject**

- Despite widespread recognition of its foundational status for the WHO Framework Convention on Tobacco Control (FCTC), implementation of Article 5.3 generally remains limited, including in many low/middle-income contexts.
- In multilevel systems, political decisions on tobacco control can occur at diverse levels of government, allowing for bottom-up innovation, though limited attention has been given to local-level approaches for enhancing FCTC implementation.

**What this paper adds**

- Policies adopted at district and state level in Karnataka highlight innovative scope for subnational approaches to managing industry interference.
- While there are encouraging signs of impact on government-industry interactions, progress is constrained by institutional tensions amid competing mandates across departments, and by tensions with national policy commitments to corporate social responsibility.
- This study illustrates the importance of moving beyond the national level to exploring local opportunities for advancing FCTC implementation and managing industry interference.

## Data Availability

Data cannot be shared openly to protect study participant anonymity.
